# Enzymatic Degradation of Zearalenone in the Gastrointestinal Tract of Pigs, Chickens, and Rainbow Trout

**DOI:** 10.3390/toxins15010048

**Published:** 2023-01-06

**Authors:** Christiane Gruber-Dorninger, Manuela Killinger, Andreas Höbartner-Gußl, Roy Rosen, Barbara Doupovec, Markus Aleschko, Heidi Schwartz-Zimmermann, Oliver Greitbauer, Zoran Marković, Marko Stanković, Karin Schöndorfer, Djuro Vukmirovic, Silvia Wein, Dian Schatzmayr

**Affiliations:** 1 DSM—BIOMIN Research Center, 3430 Tulln, Austria; 2 Institute of Bioanalytics and Agro-Metabolomics, Department of Agrobiotechnology (IFA-Tulln), University of Natural Resources and Life Sciences, Vienna, 3430 Tulln, Austria; 3 Faculty of Agriculture, Institute of Animal Sciences, University of Belgrade, 11080 Belgrade, Serbia

**Keywords:** zearalenone, hydrolase, enzyme, feed additive, gastrointestinal, degradation, chicken, swine, rainbow trout, fish

## Abstract

The estrogenic mycotoxin zearalenone (ZEN) is a common contaminant of animal feed. Effective strategies for the inactivation of ZEN in feed are required. The ZEN-degrading enzyme zearalenone hydrolase ZenA (EC 3.1.1.-, commercial name ZEN*zyme*^®^, BIOMIN Holding GmbH, Getzersdorf, Austria) converts ZEN to hydrolyzed ZEN (HZEN), thereby enabling a strong reduction in estrogenicity. In this study, we investigated the efficacy of ZenA added to feed to degrade ZEN in the gastrointestinal tract of three monogastric animal species, i.e., pigs, chickens, and rainbow trout. For each species, groups of animals received (i) feed contaminated with ZEN (chickens: 400 µg/kg, pigs: 200 µg/kg, rainbow trout: 2000 µg/kg), (ii) feed contaminated with ZEN and supplemented with ZenA, or (iii) uncontaminated feed. To investigate the fate of dietary ZEN in the gastrointestinal tract in the presence and absence of ZenA, concentrations of ZEN and ZEN metabolites were analyzed in digesta of chickens and rainbow trout and in feces of pigs. Upon ZenA administration, concentrations of ZEN were significantly decreased and concentrations of the degradation product HZEN were significantly increased in digesta/feces of each investigated animal species, indicating degradation of ZEN by ZenA in the gastrointestinal tract. Moreover, upon addition of ZenA to the diet, the concentration of the highly estrogenic ZEN metabolite α-ZEL was significantly reduced in feces of pigs. In conclusion, ZenA was effective in degrading ZEN to HZEN in the gastrointestinal tract of chickens, pigs, and rainbow trout, and counteracted formation of α-ZEL in pigs. Therefore, ZenA could find application as a ZEN-degrading feed additive for these animal species.

## 1. Introduction

The mycotoxin zearalenone (ZEN) is a frequent contaminant of animal feed [1,2,3]. As ZEN is structurally similar to the sex hormone 17β-estradiol, it binds to estrogen receptors and exerts estrogenic effects in animals [4,5]. Pigs are particularly susceptible to ZEN. ZEN can cause hyperestrogenism and impair reproductive function in this species [6,7]. Furthermore, ZEN may affect gut health and the immune system in pigs [8,9,10,11]. Poultry are more resistant to ZEN. Higher ZEN doses were reported to cause hyperestrogenism and alterations of the reproductive tract in chickens and turkeys [12,13,14]. Rainbow trout (*Oncorhynchus mykiss*) have been shown to be sensitive to dietary ZEN in recent studies. Exposure of rainbow trout to feed contaminated with ZEN at the EU guidance value (2000 µg/kg) [15] increased growth, but negatively affected reproductive health and the immune system, and increased the mortality of offspring [16,17]. Due to the detrimental effects of ZEN in pigs, poultry, and rainbow trout, efforts should be made to minimize the exposure of these animals to ZEN.

Mycotoxin contamination of cereal crops intended to be used as animal feed should be counteracted by good agricultural practices, but complete prevention of mycotoxin formation can hardly be achieved [18,19,20]. Therefore, feed additives that inactivate mycotoxins in the gastrointestinal tract are used to counteract adverse effects of mycotoxins in animals. Adsorbents that bind mycotoxins and prevent their absorption from the gastrointestinal tract (e.g., bentonite and other clay minerals) have been shown to be highly effective for removing aflatoxins, but they are less effective for removing ZEN [21,22]. The ZEN binding capacity of clays can be increased by modifying their surface structure [22,23]. However, the safety of these modified clay products is unclear [24]. Application of mycotoxin-degrading microorganisms or microbial enzymes as feed additives can be a specific, effective and irreversible method for the removal of mycotoxins from feed [25,26]. However, the activity of a mycotoxin-degrading enzyme in the gastrointestinal tract and the safety of the formed degradation products have to be carefully evaluated.

**Figure 1:**
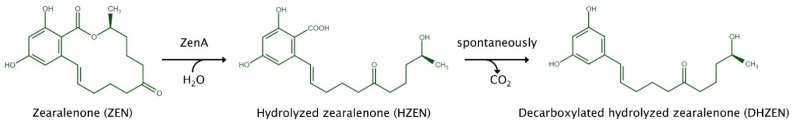
Enzymatic degradation of zearalenone by zearalenone hydrolase ZenA, modified from [29].

The potential of the enzyme zearalenone hydrolase ZenA (commercial name ZEN*zyme*^®^, BIOMIN Holding GmbH, Getzersdorf, Austria) for application as a ZEN-degrading feed additive has been investigated in previous studies. ZenA hydrolyzes the lactone ring of ZEN (Figure 1). The product of this reaction, hydrolyzed ZEN (HZEN), spontaneously converts to decarboxylated HZEN (DHZEN; Figure 1). HZEN and DHZEN were non-estrogenic in in vivo and in vitro studies. HZEN and DHZEN did not evoke an estrogenic response in an estrogen-sensitive yeast bioassay [27] or an MCF-7 cell proliferation assay [27,28]. Furthermore, in contrast to what was observed for an equimolar dietary concentration of ZEN, dietary administration of HZEN and DHZEN did not increase vulva size or uterus weight, or alter the expression of ZEN-responsive microRNAs in pigs [27]. Consequently, degradation of ZEN by ZenA enables a strong reduction in estrogenicity.

ZenA was effective as a ZEN-degrading feed additive in ruminants. ZenA degraded ZEN to HZEN in rumen fluid in vitro, thereby preventing the formation of the highly estrogenic metabolite α-zearalenol (α-ZEL) by rumen microbiota [30,31]. Moreover, when applied as a feed additive in dairy cows, ZenA readily degraded ZEN to HZEN in the rumen thereby preventing α-ZEL formation [30]. However, to date, the efficacy of ZenA as a feed additive in monogastric animal species has not been investigated.

In this study, we evaluate the efficacy of ZenA applied as a feed additive to degrade ZEN in the gastrointestinal tract of swine, chicken, and rainbow trout by investigating ZEN and its degradation products and metabolites in digesta or excreta as biomarkers. We hypothesize that ZenA degrades ZEN to HZEN in the gastrointestinal tract of these animals. This is to the best of our knowledge the first published study that investigates the efficacy of a ZEN-degrading enzyme in monogastric digestive systems.

## 2. Results

### 2.1. Efficacy of ZenA in Broiler Chickens

**Figure 2:**
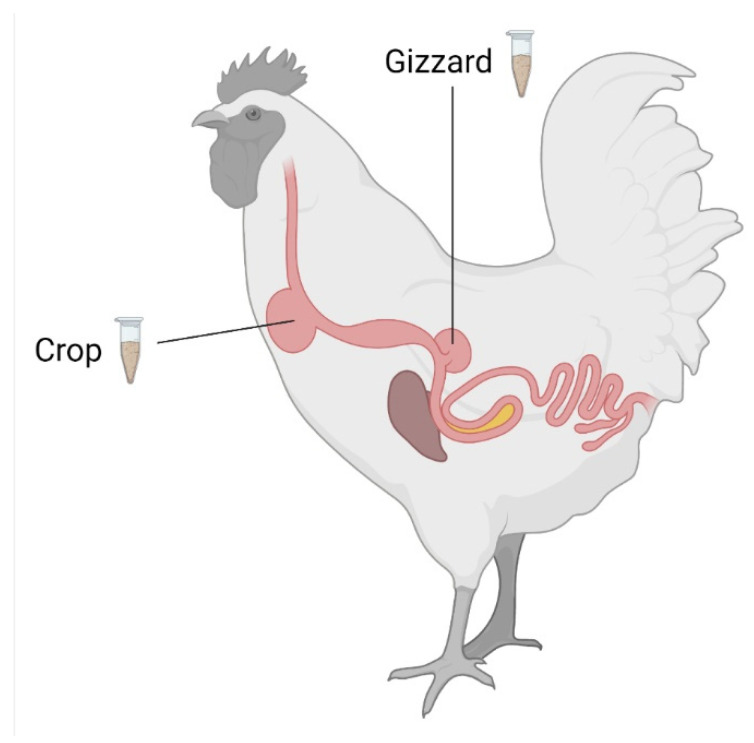
Scheme of the gastrointestinal tract of a chicken indicating sampling locations. This image was created with BioRender.com.

Knowing the minimum effective dose of ZenA is important for commercial sustainability of ZenA-based ZEN inactivation in animal feed. Here, a dose-finding trial in broiler chickens was performed. To this end, chickens received feed contaminated with ZEN (400 µg/kg) either not supplemented with ZenA (positive control), or supplemented with ZenA at an inclusion level of 5 units (U)/kg, 10 U/kg, 20 U/kg, 40 U/kg, 80 U/kg, or 160 U/kg for 14 days. One additional group of animals received feed not artificially contaminated with ZEN and not supplemented with ZenA (negative control). The dietary ZEN concentration applied in this trial was below the EU guidance value for ZEN in feed materials applicable for poultry [15] and was at the higher end of the range of ZEN levels commonly detected in animal feed [30]. To evaluate enzymatic degradation of ZEN, concentrations of ZEN, HZEN, and DHZEN were determined in crop and gizzard (Figure 2). Concentrations of ZEN and its metabolites in excreta were often below the limit of quantification (LOQ; data not shown) and were therefore not investigated further as biomarkers for ZEN degradation.

**Figure 3:**
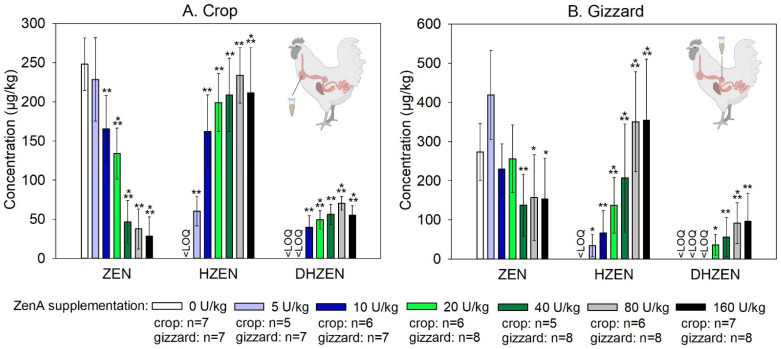
Concentrations of zearalenone and its metabolites in crop and gizzard of broiler chickens. (**A**) shows concentrations of zearalenone (ZEN), hydrolyzed ZEN (HZEN), and decarboxylated HZEN (DHZEN) in digesta obtained from the crop and (**B**) shows concentrations of ZEN, HZEN, and DHZEN in digesta obtained from the gizzard of animals that received ZEN-contaminated feed not supplemented with ZenA (positive control; white bars), or supplemented with 5 units (U) ZenA/kg feed (light blue bars), 10 U ZenA/kg feed (dark blue bars), 20 U ZenA/kg feed (light green bars), 40 U ZenA/kg feed (dark green bars), 80 U ZenA/kg feed (light grey bars), or 160 U ZenA/kg feed (black bars). Concentrations were determined in freeze-dried digesta. Bars indicate means and error bars indicate standard deviation. “<LOQ” indicates that the respective compound was below the limit of quantification in all animals of the respective treatment group. Numbers of samples analyzed per group are given in the legend. There were eight animals in each group. Sample collection was attempted from every animal, but in some cases crops or gizzards were empty at the time of sampling. Asterisks indicate statistically significant difference to positive control group (* indicates *p*-value < 0.05; ** indicates *p*-value < 0.01; *** indicates *p*-value < 0.001). For statistical analysis, data were tested for normal distribution and homogeneity of variances. Data for which normal distribution was not given were subjected to Wilcoxon’s rank sum test. Data for which normal distribution was given were subjected to a Student’s *t*-test with or without Welch’s correction for homogeneity of variances, dependent on the outcome of Levene’s test. Comparisons were made pairwise between the positive control group and each of the groups treated with ZenA. They were run 1-sided since the effect of the enzyme is unidirectional. Wilcoxon rank sum test was applied for the HZEN and DHZEN data. Student’s *t*-test was used for ZEN in crop for 5 U/kg, 10 U/kg, 20 U/kg, and 40 U/kg ZenA, and for ZEN in gizzard (all unit levels). Wilcoxon rank sum test was used for ZEN in crop for 80 U/kg and 160 U/kg ZenA. Schemes of chickens were created with BioRender.com.

Digesta obtained from the crop of animals that received ZEN-contaminated feed not supplemented with ZenA (positive control group) were found to be contaminated with ZEN, whereas HZEN and DHZEN concentrations were below the LOQ in these samples (Figure 3). ZenA supplementation of diets dose-dependently decreased the concentration of ZEN detected in the crop, and dose-dependently increased the concentration of HZEN (Figure 3). DHZEN was detected at lower concentrations than HZEN and showed a dose-dependent increase as well (Figure 3). ZenA inclusion levels of ≥10 U/kg diet significantly (*p* < 0.05) decreased ZEN concentrations in the crop compared to the positive control group (Figure 3). In animals that received uncontaminated feed not supplemented with ZenA (negative control group), only trace amounts of ZEN, HZEN, and DHZEN were detected.

In digesta obtained from the gizzard, ZEN concentrations showed a high inter-individual variation and while a dose-dependent effect of ZenA may be inferred from these data, this trend was not as clear as observed for digesta obtained from the crop (Figure 3). A significant (*p* < 0.05) reduction in ZEN concentrations in gizzard digesta compared to the positive control was observed for ZenA inclusion levels of ≥40 U/kg diet. HZEN and DHZEN concentrations in gizzard digesta increased dose-dependently with ZenA supplementation (Figure 3). In animals of the negative control group, only trace amounts of ZEN, HZEN, and DHZEN were detected in gizzard digesta.

We determined the minimum effective dose in this experiment based on ZEN concentrations detected in the crop, as ZenA had a clear dose-dependent effect on this parameter. The minimum effective dose that achieved a significant reduction of ZEN concentrations in digesta was 10 U/kg. In the gizzard, a clear dose-dependent effect on ZEN concentrations was not observed due to a higher inter-individual variability. However, dose-dependent effects of ZenA were observed on HZEN and DHZEN concentrations.

As expected with this experimental set-up, feed intake and body weight development were similar for all trial groups (Table 1).

### 2.2. Efficacy of ZenA in Pigs

We investigated the efficacy of ZenA to degrade ZEN in the gastrointestinal tract of pigs. To this end, groups of pigs received (i) feed contaminated with ZEN (200 µg/kg; positive control), (ii) feed contaminated with ZEN (200 µg/kg) and supplemented with ZenA (10 U/kg), or (iii) uncontaminated feed (negative control) for 42 days. The applied dietary ZEN concentration was in between the EU guidance value for feed destined for piglets and gilts (i.e., 100 µg/kg) and the EU guidance value for feed destined for sows and fattening pigs (i.e., 250 µg/kg) [15] and was at the higher end of the range of ZEN levels commonly detected in animal feed [30]. The ZenA concentration was selected as it was found to significantly reduce ZEN in the gastrointestinal tract of chickens (Figure 3). To evaluate the enzymatic degradation of ZEN, concentrations of ZEN, HZEN, and DHZEN were analyzed in feces. In addition, fecal concentrations of the ZEN-metabolite α-ZEL were determined.

**Figure 4:**
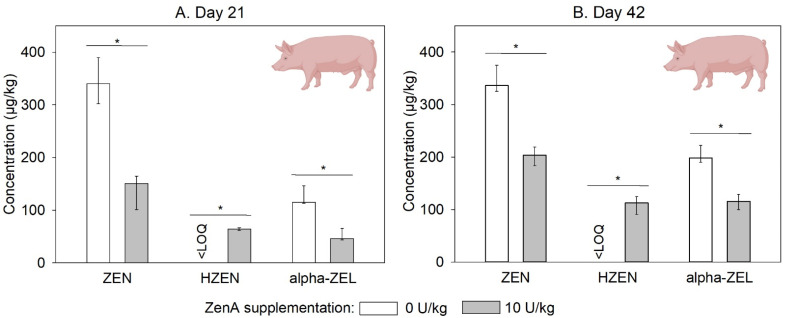
Concentrations of zearalenone and its metabolites in feces of pigs. (**A**,**B**) show concentrations of zearalenone (ZEN), hydrolyzed ZEN (HZEN), and α-zearalenol (α-ZEL) in feces collected on day 21 and day 42, respectively, from animals that received ZEN-contaminated feed not supplemented with ZenA (white bars), or ZEN-contaminated feed supplemented with 10 units (U) ZenA/kg (grey bars). Concentrations were determined in freeze-dried feces. Bars indicate medians and error bars indicate interquartile range (3 pens of 4 pigs each per treatment group; *n* = 3). “<LOQ” indicates that the respective compound was below the limit of quantification in all animals of the respective treatment group. Asterisks indicate statistically significant differences between groups (*p*-value < 0.05). Non-parametric comparisons were calculated via Wilcoxon’s rank sum test pairwise between the group that received ZEN-contaminated feed not supplemented with ZenA and the group that received ZenA. They were run one-sided since the effect of the enzyme is unidirectional. The scheme of a pig was created with BioRender.com.

In feces samples collected on days 21 and 42 of the trial, ZEN and α-ZEL concentrations were significantly lower in the group that received ZEN-contaminated feed supplemented with ZenA compared to the group that received ZEN-contaminated feed not supplemented with ZenA (Figure 4). Furthermore, HZEN was detected in feces collected from the group that received ZEN-contaminated feed supplemented with ZenA, but not in feces from the group that received ZEN-contaminated feed not supplemented with ZenA (Figure 4). DHZEN concentrations were below the LOQ in all samples. In feces of animals that received uncontaminated feed, ZEN, α-ZEL, HZEN, and DHZEN concentrations were below the LOQ.

As expected with this experimental set-up, feed intake and body weight development were similar between groups (not significantly different; data not shown). Feed intake and body weight for each group are given in Table 2.

### 2.3. Efficacy of ZenA in Rainbow Trout

We performed a feeding trial to evaluate the efficacy of ZenA to degrade ZEN in the gastrointestinal tract of rainbow trout. In this trial, groups of fish received (i) feed contaminated with ZEN (2000 µg/kg; positive control), (ii) feed contaminated with ZEN (2000 µg/kg) and supplemented with ZenA (10 U/kg), or (iii) uncontaminated feed (negative control) for 84 days. The applied dietary ZEN concentration corresponds to the EU guidance value for feed materials that is applicable for fish [15] and was at the higher end of the range of ZEN levels previously reported in aquaculture feed [32]. The applied ZenA concentration was selected as it was already shown to significantly reduce ZEN in the gastrointestinal tract of chickens and pigs (Figure 3 and Figure 4). To evaluate the enzymatic degradation of ZEN, concentrations of ZEN, HZEN, and DHZEN were determined in digesta.

**Figure 5:**
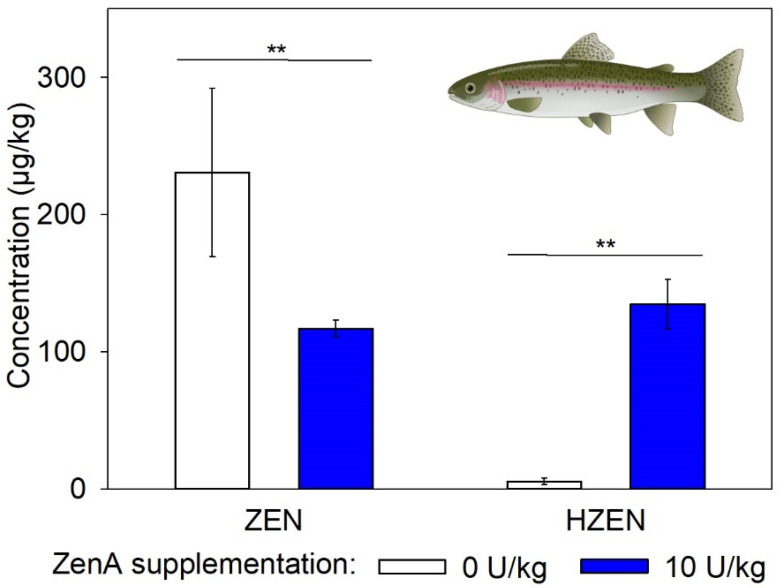
Concentrations of zearalenone and its metabolites in digesta of rainbow trout. The figure shows concentrations of zearalenone (ZEN) and hydrolyzed ZEN (HZEN) in digesta of rainbow trout that received ZEN-contaminated feed not supplemented with ZenA (positive control; white bars), or ZEN-contaminated feed supplemented with 10 units (U) ZenA/kg feed (blue bars). Concentrations were determined in freeze-dried digesta. Bars indicate means and error bars indicate standard deviation (5 pens of 8 trout each per treatment group; *n* = 5). Asterisks indicate statistically significant differences (** indicates *p*-value < 0.01). Data were tested for normal distribution and homogeneity of variances before comparing groups. Data for which normal distribution was not given were subjected to Wilcoxon’s rank sum test. Data for which normal distribution was given were subjected to the Student’s *t*-test with or without Welch’s correction for homogeneity of variances, dependent on the outcome of Levene’s test. Comparisons were made pairwise between the positive control group and the group treated with ZenA. They were run one-sided since the effect of the enzyme is unidirectional. Student’s *t*-test with Welch’s correction was used for the ZEN data, and Wilcoxon rank sum test was used for the HZEN data. The scheme of a trout was created with BioRender.com.

In digesta of fish that received a ZEN-contaminated diet supplemented with ZenA, concentrations of ZEN were significantly lower compared to fish that received a ZEN-contaminated diet without ZenA (Figure 5). Furthermore, significantly higher concentrations of HZEN were detected in digesta of fish that received ZenA (Figure 5). In case of the negative control group, 3 of 26 samples contained ZEN and HZEN concentrations in measurable quantities, while for the remaining samples concentrations were <LOQ (data not shown). DHZEN concentrations were <LOQ in samples from all groups, except for one sample of the ZenA group (data not shown).

As expected with this experimental set-up, feed intake and body weight development were similar between groups (not significantly different; data not shown). Feed intake and body weight for each group are given in Table 3.

## 3. Discussion

We investigated the efficacy of ZenA applied as a feed additive to degrade dietary ZEN in the gastrointestinal tract of chickens, pigs, and rainbow trout. In either species, administration of ZenA at a concentration of 10 U/kg significantly decreased ZEN concentrations in digesta or feces (Figure 3, Figure 4 and Figure 5). In accordance with the reaction catalyzed by the enzyme (Figure 1), HZEN appeared in digesta/feces upon administration of ZenA (Figure 3, Figure 4 and Figure 5) confirming that ZenA readily degraded ZEN to HZEN in the gastrointestinal tract. In digesta obtained from chickens, DHZEN was detected upon ZenA administration indicating spontaneous decarboxylation of HZEN during digestion. These results indicate that ZenA added to feed at an inclusion level of 10 U/kg is effective in reducing common dietary ZEN concentrations in the gastrointestinal tract of three monogastric animal species.

In chickens, a reduction in ZEN concentration and appearance of degradation products HZEN and DHZEN was already observed in the crop, indicating a degradation of ZEN during an early stage of digestion. Likewise, in dairy cows, ZEN was degraded to HZEN and DHZEN already in the rumen [30]. In the pig trial presented here, no invasive sampling was performed, and instead, biomarkers of ZEN degradation were analyzed in feces. In the trout trial, separate analysis of digesta from the proximal part of the intestines was considered. However, due to low sample volume, digesta from the entire digestive system were analyzed. Based on the observations in chickens and dairy cows, it is reasonable to hypothesize that degradation of ZEN by ZenA takes place early in the digestive process in pigs and trout as well. This could be clarified in future studies.

Pigs, particularly pre-pubertal female pigs, are known to be more sensitive to estrogenic effects of ZEN than most other species [33]. One reason for this higher sensitivity could be a high capacity for metabolization of ZEN to α-ZEL [33], a metabolite that is 60 times as estrogenic as ZEN [34]. α-ZEL and its glucuronides were the main metabolites of ZEN detected in feces [35], bile [36,37], urine [37,38], and blood [38,39] of pigs. Upon addition of ZenA to the diet of pigs, concentrations of α-ZEL were significantly reduced in feces (Figure 4), indicating that enzymatic degradation of ZEN prevented α-ZEL formation. Likewise, in dairy cows, supplementation of ZEN-contaminated feed with ZenA prevented α-ZEL formation in the rumen [30]. Therefore, while natural metabolization of ZEN in pigs and other species involves the production of α-ZEL, a compound of increased estrogenic potency, ZenA activity in the gastrointestinal tract detoxifies ZEN to HZEN, thereby reducing α-ZEL formation.

The results presented here indicate that ZenA applied as feed additive is effective in reducing ZEN concentrations in the gastrointestinal tract. However, concentrations of ZEN and metabolites in digesta or feces do not necessarily allow conclusions on internal exposure as it remains unclear to which extent these compounds are absorbed from the gastrointestinal tract. Therefore, follow-up studies will address the efficacy of feed additive ZenA to reduce the systemic exposure of animals to ZEN. This could be accomplished by analyzing concentrations of ZEN and its metabolites in blood or urine. Furthermore, to ensure that the enzyme does not exert any unintended effects, ZenA added to uncontaminated diets has to be tested in separate trials.

Zearalenone hydrolase ZenA is the first enzyme that was successfully applied as a ZEN-degrading feed additive as demonstrated here and in a previous study [30]. ZenA was furthermore effective in removing ZEN during bioethanol production in a pilot-scale experiment [40]. Other ZEN-degrading enzymes were recently evaluated for the removal of ZEN from food and feed in lab-scale experiments. Enzymes degraded ZEN in corn oil [41], as well as in wheat flour [42] and different maize products [43] upon incubation in buffer solutions. Furthermore, ZEN-degrading enzymes were expressed in transgenic plants [44,45]. These examples illustrate that enzymes that degrade mycotoxins into less toxic metabolites hold great potential for biotechnological application.

## 4. Conclusions

Results of this study indicate that the enzyme ZenA applied as a feed additive degrades ZEN to the non-estrogenic metabolite HZEN in the gastrointestinal tract of chickens, pigs, and rainbow trout. Furthermore, in pigs, ZenA reduced the formation of the highly estrogenic metabolite α-ZEL. Therefore, ZenA is effective as a ZEN-degrading feed additive in different monogastric animal species. Follow-up studies will address the efficacy of feed additive ZenA to reduce the systemic exposure of animals to ZEN, for example by analyzing concentrations of ZEN and its metabolites in blood.

## 5. Materials and Methods

### 5.1. Reagents and Standards

Acetonitrile (ACN; LC gradient grade) was purchased from VWR International GmbH (Vienna, Austria) and Chemlab (Zedelgem, Belgium). Glacial acetic acid (HAc; LC-MS grade) was obtained from Sigma-Aldrich (Vienna, Austria) and VWR (Vienna, Austria). Water was purified with a Purelab Ultra system (ELGA LabWater, Celle, Germany) or a with a Milli-Q^®^ IQ 7015 system (Merck, Darmstadt, Germany). Reference standard solutions of ZEN (100.8 µg/mL in ACN) and α-ZEL (10.4 µg/mL in ACN) were obtained from Romer Labs (Tulln, Austria). HZEN and DHZEN standards (purity > 95%) were produced as described by Vekiru and coworkers [29].

### 5.2. Broiler Chicken Feeding Trial

#### 5.2.1. Experimental Setup

A feeding trial with broiler chickens (*Gallus gallus domesticus*) was performed at the Center for Applied Animal Nutrition (CAN) in Nitzing, Austria. One-day-old broiler chickens (Ross 308; mixed sex) were obtained from a local producer. Animals were kept in an environmentally controlled poultry house on wood shavings. Climate conditions and light/dark cycles were regulated according to the breeding company’s recommendations. Pens were equipped with feeders and bell shape drinkers with a nipple. Feed and water were available ad libitum.

All procedures related to these experiments were performed according to Austrian law and following the European Guidelines for the Care and Use of Animals for Research Purpose [46]. The animal experiment was approved by Office of the Federal Government of Lower Austria (LF1-TVG-39/039-2016). Qualified personnel monitored the general clinical status of the chickens twice a day. All incidences were recorded daily. No medication was administered, and no mortalities occurred.

Following a 14-day acclimation period, chickens (~400 g) were allocated to 8 treatment groups (1 pen per group, 8 animals per pen) taking into consideration the body weight of the animals. During a 14-day feeding trial, treatment groups received (i) basal feed, (ii) ZEN-contaminated feed (400 µg ZEN/kg feed), (iii) ZEN-contaminated feed supplemented with 5 U/kg ZenA, (iv) ZEN-contaminated feed supplemented with 10 U/kg ZenA, (v) ZEN-contaminated feed supplemented with 20 U/kg ZenA, (vi) ZEN-contaminated feed supplemented with 40 U/kg ZenA, (vii) ZEN-contaminated feed supplemented with 80 U/kg ZenA, or (viii) ZEN-contaminated feed supplemented with 160 U/kg ZenA. Researchers that fed the animals, checked the status of the animals, or analyzed samples were blinded to the group allocation of the animals. Since animals were kept in pens for animal welfare reasons, and animals had to be sacrificed at the end of the trial, and in order to minimize the number of animals in this trial, one pen per group was used, and the individual animal was considered the experimental unit.

#### 5.2.2. Diet

The basal diet consisted of a mash feed based on maize and soy as specified in Table 4.

Concentrations of ZEN, aflatoxin B1, deoxynivalenol, T-2 toxin, fumonisin B1, and ochratoxin A in basal feed were analyzed at the Department of Agrobiotechnology (IFA-Tulln) at the University of Natural Resources and Life Sciences Vienna (BOKU) in Tulln, Austria according to the method described in [47]. Concentrations of ZEN, deoxynivalenol, and fumonisin B1 were 30.3 µg/kg, 177 µg/kg, and 265 µg/kg, respectively. Concentrations of aflatoxin B1, T-2 toxin, and ochratoxin A were below the limit of detection.

#### 5.2.3. Preparation of ZEN-Contaminated and ZenA-Supplemented Feed

For the preparation of ZEN-contaminated feed, ZEN lyophilizate with maltodextrin as carrier was produced as described previously [30] and mixed into the feed at an inclusion level of 0.1%. For the preparation of ZEN-contaminated feed supplemented with different levels of ZenA, a ZenA preparation (ZEN*zyme*^®^; EC 3.1.1.-; BIOMIN Holding GmbH, Getzersdorf, Austria) was mixed into ZEN-contaminated feed to achieve an activity of 5, 10, 20, 40, 80, or 160 U/kg. In ZEN-contaminated feed and in the ZEN-contaminated diets supplemented with different levels of ZenA, a final ZEN concentration of ~400 µg/kg was verified by HPLC-MS/MS analysis. To this end, ZEN was extracted from feed as described previously [30]. HPLC-MS/MS analysis is described below (Section 5.2.5). Measured ZEN concentrations in feed were 37 µg/kg, 417 µg/kg, 515 µg/kg, 420 µg/kg, 423 µg/kg, 491 µg/kg, 479 µg/kg, and 457 µg/kg for the negative control group, the positive control group, and the groups that received 5 U/kg, 10 U/kg, 20 U/kg, 40 U/kg, 80 U/kg, and 160 U/kg of ZenA, respectively.

#### 5.2.4. Sampling and Sample Preparation

On day 14 of the trial, all animals were sacrificed by administration of an overdose of carbon dioxide. Digesta contents from crop and gizzard were removed, immediately placed on ice, subsequently stored at −20 °C, and subsequently lyophilized.

For analysis of ZEN and its degradation products, 1 g of each lyophilized digesta sample was extracted with 15 mL ACN/water (80/20, *v*/*v*) in a 50 mL reaction tube on a rotary shaker at room temperature for 30 min. Subsequently, the tube was centrifuged for 10 min at 2300× *g*, and the supernatant was transferred to a clean 50 mL reaction tube. The digesta sample was again extracted and centrifuged in the same way. The supernatants resulting from both extraction procedures were combined, and 1 mL of the combined supernatant was transferred to a clean reaction tube and centrifuged for 5 min at 2300× *g*. The resulting supernatant was stored at −20 °C until HPLC-MS/MS analysis (Section 5.2.5).

#### 5.2.5. Analysis of ZEN and its Metabolites in Digesta and Analysis of ZEN in Feed

ZEN and its metabolites in digesta and feed were analyzed on a 1290 Infinity series UHPLC system (Agilent Technologies, Waldbronn, Germany) coupled to a 6500+ QTrap mass spectrometer equipped with an IonDrive TurboV source (Sciex, Foster City, CA, USA). Chromatographic separation was performed on a Kinetex C18 column (150 mm × 2.1 mm, 2.6 µm, Phenomenex, Aschaffenburg, Germany). Mobile phase A was composed of water/HAc (A = 99.9/0.1, *v*/*v*), and mobile phase B consisted of ACN/HAc (99.9/0.1, vol/vol). The total run time was 20 min. The gradient started at 15% B, which was held for 0.5 min. Thereafter, the proportion of B was linearly increased to 60% at 13.5 min, and to 100% at 14 min. Subsequently, 100% B was held until 16.9 min. Finally, the column was re-equilibrated at 15% B for 3 min. Injection volume, flow rate, and column temperature were 2 µL, 250 µL/min, and 30 °C, respectively. Mass spectrometric detection was carried out in negative electrospray ionization mode with selected reaction monitoring as scan type (Table 5). The following source settings were applied: ion spray voltage −4500 V, source temperature 400 °C, curtain gas 35 psi, ion source gas 1 at 60 psi, and gas 2 at 40 psi. LOQs for ZEN, HZEN, and DHZEN in chicken digesta were 30 ng/g (94 nmol/kg), 30 ng/g (89 nmol/kg), and 30 ng/g (103 nmol/kg), respectively. LOQs for ZEN, HZEN, DHZEN, and α-ZEL in pig feces were 40 ng/g (126 nmol/kg), 47 ng/g (140 nmol/kg), 174 ng/g (596 nmol/kg), and 53 ng/g (166 nmol/kg), respectively. The LOQ for ZEN in chicken and pig feed was 30 ng/g (94 nmol/kg).

### 5.3. Pig Feeding Trial

#### 5.3.1. Experimental Setup

A feeding trial with pigs (*Sus scrofa domesticus*) was performed at the pig facility of the Center for Applied Animal Nutrition (CAN) in Mank, Austria. In total, 36 pigs (~10 kg; 4 weeks old; Austrian genotype Ö-HYB-F1 [(Landrace × Large White) × Pietrain] were obtained from a local producer. All animals were ear-tagged for individual identification. Animals were kept in pens with slatted floors equipped with cup drinkers and feed troughs. Feed and water were provided ad libitum. Climate conditions and lighting program were regulated automatically according to standard recommendations for weaning piglets. Due to the presence of windows on both sides of the stable, light/dark cycles corresponded to actual diurnal cycles.

Following a 12-day acclimation period, pigs were allocated to 3 treatment groups taking into consideration the body weight and sex of the animals (3 pens per group, 2 female and 2 male pigs per pen). During a 42-day feeding trial, treatment groups received (i) basal feed, (ii) ZEN-contaminated feed (200 µg ZEN/kg feed), or (iii) ZEN-contaminated feed (200 µg ZEN/kg feed) supplemented with 10 U/kg ZenA. The pen was considered the experimental unit. Researchers that fed the animals, checked the status of the animals, or analyzed samples were blinded to the group allocation of the animals.

This feeding trial was approved by Office of the Federal Government of Lower Austria (LF1-TVG-39/042-2017) and was performed according to Austrian law and following the European Guidelines for the Care and Use of Animals for Research Purpose [46]. Qualified personnel monitored and documented the general clinical status of the pigs daily. No medication was necessary and no mortalities occurred.

#### 5.3.2. Diet

Piglets received a grower diet consisting of a mash feed based on wheat, soy, and barley as specified in Table 6.

The diet was analyzed for the most relevant mycotoxins, i.e., ZEN, deoxynivalenol, ochratoxin A, fumonisins B1 and B2, as well as aflatoxins B1, B2, G1, and G2 by Romer Labs GmbH (Tulln, Austria) using HPLC-MS/MS analysis. Concentrations of all mycotoxins were below the limit of detection.

#### 5.3.3. Preparation of ZEN-Contaminated and ZenA-Supplemented Feed

For the preparation of ZEN-contaminated feed, a blend of culture material of *Fusarium graminearum* (obtained from Romer Labs GmbH, Tulln, Austria) and inulin was mixed into the feed at an inclusion level of 0.11%. For the preparation of ZEN-contaminated feed supplemented with ZenA, a ZenA preparation (ZEN*zyme*^®^; EC 3.1.1.-; BIOMIN Holding GmbH, Getzersdorf, Austria) was mixed into ZEN-contaminated feed to achieve an activity of 10 U/kg. A final ZEN concentration of ~200 µg/kg in ZEN-contaminated feed and ZEN-contaminated feed supplemented with ZenA was verified by HPLC-MS/MS analysis. To this end, ZEN was extracted from feed as described previously [30]. HPLC-MS/MS analysis was performed as described above (Section 5.2.5). Measured ZEN concentrations in feed were <LOQ for the negative control group, 222 µg/kg for the positive control group and 191 µg/kg for the group that received ZenA.

#### 5.3.4. Sampling and Sample Preparation

On days 21 and 42 of the trial, feces samples were collected from each individual animal and lyophilized. For analysis of ZEN and its metabolites, each lyophilized feces sample was homogenized in a plastic bag. Homogenized feces samples were extracted as described by Binder and coworkers [35].

#### 5.3.5. Analysis of ZEN, HZEN, DHZEN, and α-ZEL in Feces

HPLC-MS/MS analysis was performed as described in Section 5.2.5.

### 5.4. Fish Feeding Trial

#### 5.4.1. Experimental Setup

A feeding trial with juvenile rainbow trout (*Oncorhynchus mykiss*; ~2 months old, mean weight: 9.79 g) was conducted in the Laboratory for Fish Nutrition of the Institute of Animal Science, Faculty of Agriculture, University of Belgrade. The research was conducted under approval issued by the Ministry of Agriculture, Forestry and Water Management, Republic of Serbia (number: 323-07-13151/2021-5) in accordance with Serbian law and following the European Guidelines for the Care and Use of Animals for Research Purpose [46]. All animal procedures were approved by the Ethical Committee for the Use of Laboratory Animals of the University of Belgrade, Faculty of Agriculture.

Fish originated from a selective breeding program of the Centre for Fishery and Applied Hydrobiology (CEFAH), Faculty of Agriculture, University of Belgrade. In total, 300 fish were transferred to the Laboratory for Fish Nutrition. After transport, fish were randomly allocated to 15 individual flow-through tanks of 120 L each (20 fish per tank). Thereafter, during an adaptation period of 18 days, fish received commercial feed (Skretting Pro Aqua, 1.5 mm, see Section 5.3.2). Following the adaptation period, the feeding trial was performed for a period of 12 weeks (84 days). Groups of fish (5 tanks per group) received (i) basal feed, (ii) ZEN-contaminated feed (2000 µg ZEN/kg feed), or (iii) ZEN-contaminated feed (2000 µg/kg ZEN/kg feed) supplemented with 10 U/kg ZenA. The tank was considered the experimental unit.

Water supply consisted of continuous flow dechlorinated municipal tap water at the rate of 0.34 L/min. Each tank was equipped with a belt feeder device (AGK Kronawitter, Wallersdorf, Germany), and fish were fed at a rate of 2% of feed per day (The weight of the feed given to fish was equivalent to 2% of the animal’s body weight. The ration was adjusted weekly according to the theoretical growth curve, and every 4 weeks the curve was corrected based on the measured weight). Once per day, all tanks were inspected by qualified personnel. Health and behavior of fish was monitored, and mortalities were recorded (one fish died during the trial from a skin lesion, which was most likely not related to the experiment). Once per day, feed was weighed and placed on belt feeders. Water quality was monitored by daily measurement of oxygen concentration, oxygen saturation, temperature, pH, and electroconductivity using a MULTI 340i/SET apparatus (WTW, Germany). Furthermore, concentrations of nitrogen compounds in the water (ammonia, nitrite, nitrate) were determined once every 2 weeks. These analyses were conducted in the Laboratory for Water Quality of the Faculty of Agriculture, University of Belgrade.

#### 5.4.2. Diet

The experimental diets were based on commercial premium rainbow trout feedstuffs that were modified by post-pellet coating (see Section 5.3.3). For the basal diets, 2 pellet sizes with different feed formulas were chosen (Skretting Pro Aqua, 1.5 mm and Skretting Optiline 2.5 mm; Skretting, Stavanger, Norway), in line with recommendations of the feed manufacturer for the experimental fish body weight. Diet composition as declared by the commercial producer was based on animal protein (feed ingredients: poultry protein, soy meal feed, faba beans, hydrolyzed feather protein, wheat, poultry fat, fish meal, rapeseed oil, swine hemoglobin powder, fish oil, and wheat gluten; Table 7).

#### 5.4.3. Preparation of ZEN-Contaminated and ZenA-Supplemented Feed

ZEN and ZenA were applied to the feed by post-pellet coating using a modified version of the “pan coating” method described by Dobsikova et al. [48] for preparation of medicated feed pellets. In short, feed pellets were first coated with adhered sorbent (silica) and were subsequently coated with an aqueous dispersion of active ingredients. The coating procedure involved three consecutive steps as described below:

(A) In a first step, sorbent was added to the pellets. To this end, 2928 g pellets were placed in a drum mixer (63 l, 27.5 rpm; VidaXL, Limburg, The Netherlands), and rotation was started. In total, 100 g of colloidal silica (Aerosil^®^ 200; Evonik Operations GmbH, Essen, Germany) were gradually added to the rotating pellets, and the mixture was stirred for 3 min until a uniform adhesive layer formed on the pellet surface.
(B) For each experimental group, a separate coating dispersion was prepared.

For the production of the coating dispersion to be applied to control feed, 30 g of commercial corn meal (ZEN content: 42.2 µg/kg; particle size distribution: 90% of particles < 300 µM) and 42 g of pregelatinized corn starch (Starch^®^ 1500; Colorcon Limited, Dartford, UK) were gradually added to 270 g of pre-heated (30 °C) purified water while mixing at a stirring speed of 1000 rpm using a turbine mixer (Rührwerk RZR 2020; Heidolph, Schwabach, Germany). Subsequently, the mixture was stirred for another 15 min under the same conditions.

The coating dispersion for the production of ZEN-contaminated feed was prepared as described for control feed with the following modification. Instead of a 30 g commercial corn meal, 30 g of ZEN-containing corn meal-based culture material of *Fusarium graminearum* (corn meal media 0.354 g/kg; particle size distribution: 90% of particles < 300 µM) mixed with commercial corn meal at a 1.3:1 ratio was used. This modification enabled a final ZEN concentration in the feed of ~2000 µg/kg, while maintaining the same corn meal addition rate as for the control feed.

The coating dispersion for the production of ZEN-contaminated and ZenA-supplemented feed was prepared as described for ZEN-contaminated feed with the following modification. In total, 5 mL of purified water was replaced with an equivalent volume of purified water containing ZenA (ZEN*zyme*^®^; EC 3.1.1.-; BIOMIN Holding GmbH, Getzersdorf, Austria) to achieve a final enzyme activity of 10 U/kg feed.

(C) For pan coating, the coating dispersion (B) was gradually poured onto the pellets with adhered sorbent (A) over a period of 10 min. After coating, pellets were distributed in plastic trays in 2 cm layers and dried in a laboratory incubator (Binder BD 240; Binder GmbH, Tuttlingen, Germany) with fan-assisted forced air circulation for 24 h. Samples were dried at 45 °C with 60 rpm fan speed and completely opened an air flap to maximize air exchange.

Concentrations of common mycotoxins were determined in control feed as described previously [49]. In 1.5 mm pellets, ZEN and deoxynivalenol were detected at low concentrations (16.16 µg/kg and 14.10 µg/kg, respectively), while aflatoxin B1, T-2 toxin, fumonisin B1, and ochratoxin A were below the detection limit. In 2.5 mm pellets, a very low concentration of ZEN was detected (0.22 µg/kg), while deoxynivalenol, aflatoxin B1, T-2 toxin, fumonisin B1, and ochratoxin A were below the detection limit. Final ZEN concentrations of ~2000 µg/kg in ZEN-contaminated feed were verified by HPLC-MS/MS analysis (as described in Section 5.2.3 and Section 5.2.5). Measured ZEN concentrations in feed were 2396 µg/kg and 1648 µg/kg in case of 1.5 mm pellets and 2.5 mm pellets, respectively.

#### 5.4.4. Sampling and Sample Preparation

On day 84 of the trial, all fish were anesthetized and subsequently euthanized by immersion in over-dosed clove oil bath (0.02%). Digesta contents from the proximal and distal gastrointestinal tract were removed, immediately placed on ice, subsequently stored at −20 °C, and subsequently lyophilized.

For analysis of ZEN and its degradation products, 100 mg of each lyophilized digesta sample was spiked with a stable isotope-labelled internal standard and extracted with 1 mL of ACN/water (50/50, *v*/*v*) by shaking for 60 min on a horizontal plate shaker. Subsequently, the tube was centrifuged at 2300× *g* for 10 min, and the supernatant was transferred to a fresh 5 mL Eppendorf tube.

Two more extraction steps were performed by resuspending the pellet in 1 mL of ACN/water (50/50, *v*/*v*) by vortexing and then shaking for 30 min, followed by a centrifugation step at 2300× *g* (second extraction) or 19,000× *g* (third extraction) for 10 min. All three supernatants were pooled, vortexed briefly, and 400 µL of the extract was transferred to a fresh 1.5 mL Eppendorf tube. Subsequently, 600 µL of ethyl acetate were added and the tubes vortexed for 20 s before centrifugation at 19,083× *g* for 5 min. The upper (organic) phase was transferred to a new 2 mL Eppendorf tube. The aqueous residue was acidified with 5 µL of HAc, 600 µL of ethyl acetate was added and the samples vortexed for 10 s. After centrifugation at 19,083× *g* for 5 min, the upper phases were combined and evaporated to dryness in a sample concentrator. The dried residue was reconstituted in 400 µL of ACN/water (50/50, *v*/*v*), vortexed for 10 min and centrifuged at 19,083× *g* for 5 min. In total, 200 µL of supernatant was transferred into an HPLC vial with insert for analysis and measured using HPLC-MS/MS, or stored at 4 °C for a maximum of 24 h.

#### 5.4.5. Analysis of ZEN, HZEN, and DHZEN in Digesta

ZEN, HZEN, and DHZEN in digesta were analyzed on a 1290 Infinity series UHPLC system (Agilent Technologies, Waldbronn, Germany) coupled to a Sciex 5500 QTRAP mass spectrometer (Sciex, Foster City, CA, USA). Chromatographic separation was performed on a Kinetex EVO C18 column (150 mm × 2.1 mm, 2.6 µm, Phenomenex, Aschaffenburg, Germany). Eluents A and B consisted of water/ACN/HAc (A = 94.9/5.0/0.1, vol/vol/vol; B = 4.9/95.0/0.1, *v*/*v*/*v*). The total run time was 3.95 min. The gradient started at 38% B, which was held for 0.2 min. Thereafter, the proportion of B was linearly increased to 55% at 2.6 min, and to 100% at 2.7 min. Subsequently, 100% B was held until 3.4 min. Finally, the column was re-equilibrated at 38% B for 0.45 min. Injection volume, flow rate and column temperature were 1 µL, 0.65 mL/min and 30 °C, respectively. Mass spectrometric detection was carried out in negative electrospray ionization mode with multiple reaction monitoring as scan type. The following source settings were applied: ion spray voltage −4500 V, source temperature 500 °C, curtain gas 35 psi, ion source gas 1 at 50 psi, and ion source gas 2 at 50 psi. LOQs for ZEN, HZEN, and DHZEN were 28 ng/g (88 nmol/kg), 28 ng/g (83 nmol/kg), and 70 ng/g (240 nmol/kg), respectively.

### 5.5. Statistical Analysis

Statistics were run in R software (R Core Team, R Foundation for Statistical Computing, Vienna, Austria) version 4.0.3 in RStudio version 1.4.1106, additionally using the tidyverse package version 1.3.1 for data wrangling, the car package version 3.0–12 for Levene’s test for homogeneity of variances and the pastecs package version 1.3.21 for descriptive statistics and the Shapiro–Wilk test for normality.

For the chicken and rainbow trout trials, data were tested for normal distribution and homogeneity of variances before comparing groups. Data for which normal distribution was not given were subjected to Wilcoxon’s rank sum test. Data for which normal distribution was given were subjected to a Student’s *t*-test with or without Welch’s correction for homogeneity of variances, dependent on the outcome of Levene’s test. Comparisons were made pairwise between the positive control group and each of the groups that were treated with ZenA. They were run 1-sided since the effect of the enzyme is unidirectional (decrease of ZEN; increase of HZEN and DHZEN). For the rainbow trout trial, Student’s *t*-test with Welch’s correction was used for the ZEN data, and Wilcoxon rank sum test was used for the HZEN data. For the chicken trial, Wilcoxon rank sum test was used for the HZEN and DHZEN data. Furthermore, Student’s *t*-test was used for ZEN in crop for 5 U/kg, 10 U/kg, 20 U/kg, and 40 U/kg ZenA, and for ZEN in gizzard (all unit levels). Wilcoxon rank sum test was used for ZEN in crop for 80 U/kg and 160 U/kg ZenA.

For the pig trial, non-parametric comparisons were calculated via Wilcoxon’s rank sum test without prior testing for normal distribution and homogeneity of variances due to the smaller sample size (three replicate pens). Comparisons were run 1-sided.

If the concentration of ZEN, HZEN, DHZEN, or α-ZEL in a sample was below the LOQ, the concentration was assumed to be LOQ/2 for calculation of mean and standard deviation and for statistical analysis.

## Figures and Tables

**Table 1:** Feed intake and body weight development of broiler chickens.

Treatment Group ^1^	Total Feed Intake per Animal (Average)	Weight per Pen at Trial Start	Weight per Pen at Trial End
Negative control	1.31 kg	3.22 kg	9.70 kg
Positive control	1.28 kg	3.17 kg	9.70 kg
5 U/kg ZenA	1.31 kg	3.18 kg	9.62 kg
10 U/kg ZenA	1.37 kg	3.17 kg	9.48 kg
20 U/kg ZenA	1.31 kg	3.15 kg	9.40 kg
40 U/kg ZenA	1.33 kg	3.13 kg	9.69 kg
80 U/kg ZenA	1.24 kg	3.17 kg	9.22 kg
160 U/kg ZenA	1.35 kg	3.14 kg	9.99 kg

^1^ Negative control group received basal feed. Positive control group received feed contaminated with 400 µg/kg zearalenone. ZenA groups received feed contaminated with 400 µg/kg zearalenone and supplemented with ZenA at the given level.

**Table 2:** Feed intake and body weight development of pigs.

Treatment Group ^1^	Total Feed Intake per Animal (Average)	Weight per Animal at Trial Start (Average)	Weight per Animal at Trial End (Average)
Negative control	45.85 kg	9.21 kg	34.85 kg
Positive control	46.16 kg	9.21 kg	34.95 kg
ZenA	46.30 kg	9.21 kg	35.60 kg

^1^ Negative control group received basal feed. Positive control group received feed contaminated with 200 µg/kg zearalenone. ZenA group received feed contaminated with 200 µg/kg zearalenone and supplemented with ZenA.

**Table 3:** Feed intake and body weight development of rainbow trout.

Treatment Group ^1^	Feed Intake per Animal per Day (Average)	Weight per Animal at Trial Start (Average)	Weight per Animal at Trial end (Average)
Negative control	0.49 g	9.8 g	50.5 g
Positive control	0.48 g	9.8 g	50.9 g
ZenA	0.48 g	9.8 g	51.3 g

^1^ Negative control group received basal feed. Positive control group received feed contaminated with 2000 µg/kg zearalenone. ZenA group received feed contaminated with 2000 µg/kg zearalenone and supplemented with ZenA.

**Table 4:** Diet composition chicken trial.

Ingredients	%
Maize	62.00
Soy 48%	23.80
Full-fat soy	5.55
Sunflower oil	2.00
Monocalcium phosphate	1.70
Calcium carbonate	1.55
Fat powder	1.50
Pumpkin cake	0.58
Sodium bicarbonate	0.28
L-Lysine	0.27
DL-Methionine	0.20
Sodium chloride	0.18
Magnesium phosphate	0.10
L-Threonine	0.10
Cholinchloride	0.07
Vitamin and trace element premix ^1^	0.12
**Analyzed composition**	
Dry matter (%)	88.1
Crude protein (%)	18.3
Crude fiber (%)	2.6
Crude fat (%)	7.5
Neutral detergent fiber (%)	8.7
Non-fiber carbohydrates (%)	47.5
N-free extract (%)	53.6
Starch (%)	38.2
Crude ash (%)	6.1
Metabolizable energy (MJ)	11.83

^1^ Concentration of ingredients given per kg complete diet: iron 3.6 mg, copper 3.6 mg, zinc 24 mg, manganese 26.4 mg, iodine 0.288 mg, selenium 0.096 mg, vitamin A 2400 IE, vitamin D3 1200 IE, vitamin E 14.4 mg, vitamin K3 0.96 mg, vitamin B1 0.72 mg, vitamin B2 1.92 mg, vitamin B6 1.2 mg, vitamin B12 4.8 mg, calcium-D-pantothenate 4.8 mg, niacinamide 16.8 mg, folic acid 0.528 mg, biotin 52.8 mg, choline chloride 48 mg, betaine 22.152 mg, lysin 0.00012%, calcium 0.02868%.

**Table 5:** Mass transitions and MS parameters.

Analyte ^1^	Q1 Mass (m/z)	Q3 Mass (m/z) ^2^	Declustering Potential (V)	Collision Energy (V) ^2^
ZEN	317.1	131.0/175.0	−120	−42/−34
α-ZEL	319.1	275.1/160.0	−125	−30/−42
HZEN	335.0	149.0/161.0	−100	−34/−34
DHZEN	291.1	149.1/161.1	−100	−25/−25

^1^ Abbreviations: ZEN—zearalenone; α-ZEL—α-zearalenol; HZEN—hydrolyzed zearalenone; DHZEN—decarboxylated HZEN. ^2^ Quant/qual.

**Table 6:** Diet composition pig trial.

Ingredients	%
Wheat	60.00
Soy 48%	20.00
Barley	14.00
Calcium carbonate	1.20
Wheat bran	1.00
Monocalcium phosphate	0.90
Vinasse	0.80
Sodium chloride	0.45
Rye bran	0.40
L-Lysine	0.25
Rapeseed oil	0.20
Magnesium phosphate	0.20
DL-Methionine	0.19
L-Threonine	0.10
Tryptophan	0.07
Trace element and Vitamin premix ^1^	0.30
**Analyzed composition**	
Dry matter (%)	87.4
Crude protein (%)	16.9
Crude fat (%)	6.4
Crude fiber (%)	3.0
N-free extract (%)	53.5
Crude ash (%)	7.4
Starch (%)	35.8
Metabolizable energy (MJ)	13.37

^1^ Concentration of ingredients given per kg complete diet: lysine 0.033%, methionine 0.0099%, threonine 0.0165%, tryptophan 0.0006%, calcium 0.021%, phosphorus 0.00195%, sodium 0.0003%, magnesium 0.0039%, iron 9.3 mg, copper 6.75 mg, zinc 9 mg, manganese 6 mg, iodine 0.225 mg, selenium 0.03 mg, vitamin A 1200 IE, vitamin D3 150 IE, vitamin E 11.25 mg, vitamin K3 0.3 mg, vitamin B1 0.21 mg, vitamin B2 0.615 mg, vitamin B6 0.375 mg, vitamin B12 3.75 mg, calcium-D-pantothenate 1.5 mg, niacinamide 4.5 mg, folic acid 0.078 mg, biotin 11.25 mg, choline chloride 37.5 mg.

**Table 7:** Composition of rainbow trout diets.

**Analyzed composition of 1.5 mm pellets**	
Dry matter (g/kg)	939
Crude protein (g/kg)	469
Crude fat (g/kg)	229
Crude fiber (g/kg)	6
Crude ash (g/kg)	61
Starch (g/kg)	145
**Analyzed composition of 2.5 mm pellets**	
Dry matter (g/kg)	929
Crude protein (g/kg)	589
Crude fat (g/kg)	140
Crude fiber (g/kg)	6
Crude ash (g/kg)	85
Starch (g/kg)	105

## Data Availability

Data are contained within the article.
